# Climate‐Sensitive Transmission of Human Brucellosis: A Systematic Review of Climatic and Environmental Determinants in the Middle East

**DOI:** 10.1155/jotm/8884033

**Published:** 2026-08-30

**Authors:** Aboozar Soltani, Kourosh Azizi, Mohammad Ebrahimi, Marziyeh Hamyali-Ainvand

**Affiliations:** ^1^ Department of Medical Entomology and Vector Control, School of Health, Research Center for Health Sciences, Institute of Health, Shiraz University of Medical Sciences, Shiraz, Iran, sums.ac.ir; ^2^ Student Research Committee, Shiraz University of Medical Sciences, Shiraz, Iran, sums.ac.ir

**Keywords:** climate change, environmental determinants, human brucellosis, Middle East, one health, zoonotic diseases

## Abstract

**Background:**

Human brucellosis (HB) is a climate‐sensitive zoonotic disease whose transmission is influenced by complex interactions among climatic, environmental, livestock, and human behavioral factors. Although the Middle East is a major climate change hotspot and remains highly endemic for HB, evidence regarding climate–brucellosis relationships has not previously been systematically synthesized. This review aimed to evaluate the influence of climatic and environmental determinants on HB across the Middle East.

**Methods:**

This systematic review was conducted in accordance with the PRISMA 2020 guidelines and prospectively registered in the Open Science Framework. PubMed, Embase, Scopus, and Web of Science were systematically searched from database inception to February 9, 2025. Studies examining the association between climatic or environmental factors and HB in Middle Eastern countries were included. Owing to substantial methodological heterogeneity, findings were synthesized using a structured narrative approach.

**Results:**

Thirteen studies met the inclusion criteria. Temperature and precipitation were each investigated in nine studies, whereas humidity was examined in five studies. Temperature demonstrated both positive and negative associations with HB incidence across different climatic settings, indicating context‐dependent effects influenced by regional climate, ecological characteristics, and livestock management practices. Precipitation and humidity also showed heterogeneous associations, while wind speed consistently demonstrated a positive indirect association with disease occurrence. Seasonal analyses identified higher HB incidence during spring and summer, and vegetation cover was consistently associated with increased disease risk. Considerable heterogeneity was observed in study design, climatic settings, statistical methods, and adjustment for potential confounding factors. Because most included studies were observational, the reported associations should not be interpreted as evidence of direct causality.

**Conclusions:**

Climatic and environmental factors appear to play an important role in shaping HB transmission through interconnected human–animal–environment pathways in the Middle East. However, these relationships are highly context‐dependent and influenced by ecological conditions, livestock management systems, and public health factors. Future multinational studies integrating climatic, environmental, veterinary, and epidemiological data within a One Health framework are needed to strengthen climate‐informed surveillance, early warning systems, and brucellosis prevention strategies across the region.

## 1. Introduction

Brucellosis is a zoonotic bacterial disease affecting both animals and humans [1, 2]. Also known as “Mediterranean flaccid fever” or “Maltese fever,” it is caused by three main *Brucella* species—*B. abortus*, *B. melitensis*, and *B. suis*—that are pathogenic to humans and endemic in many countries [3, 4]. While brucellosis poses a serious threat to livestock, its impact on human health is often overlooked and remains a neglected issue in many regions [5, 6]. In humans, the disease resembles influenza, causing fever, sweating, and fatigue, and can lead to chronic complications like arthritis, myocarditis, and neuropathy. Transmission occurs through consumption of unpasteurized dairy products or contact with infected animal tissues, putting livestock farmers, slaughterhouse workers, and veterinarians in endemic areas at risk. Beyond its clinical and occupational dimensions, human brucellosis (HB) is increasingly recognized as an environmentally sensitive zoonosis, as the survival of Brucella spp. Outside the host and the intensity of human–animal contact are strongly shaped by local climatic and ecological conditions [4, 6, 7].

Although HB is globally distributed, its occurrence is strongly shaped by the interaction between climatic conditions and socioenvironmental contexts [8, 9]. Rather than acting as a direct cause of infection, climatic variability influences HB through indirect and mediated pathways. These include changes in livestock reproductive cycles, grazing behavior, pathogen survival in the environment, and the frequency of human–animal interactions, all of which collectively shape transmission dynamics [10, 11]. Climatic variables such as temperature, humidity, precipitation, and wind patterns influence the environmental persistence of Brucella spp. and modulate transmission intensity, often with delayed effects on human incidence. Several studies have reported lag periods of approximately two to four months between climatic exposure and reported human cases, reflecting the indirect pathways through which climate operates, including livestock reproduction cycles, grazing behavior, and human–animal interactions [12–20]. In the context of climate change, alterations in temperature and hydrometeorological patterns further amplify these dynamics by reshaping ecological systems and exposure pathways, thereby increasing the risk of brucellosis transmission in vulnerable populations [10, 18, 21–23].

The Middle East, particularly the Eastern Mediterranean and Middle East (EMME) region, is recognized as a major climate change hotspot, experiencing warming at nearly twice the global average rate, alongside increasing frequency and intensity of droughts and growing ecological instability [24, 25]. Climate‐sensitive diseases are conditions whose occurrence, transmission dynamics, or geographical distribution are influenced by climatic variability through direct or indirect effects on pathogens, hosts, reservoirs, vectors, or environmental conditions. HB is considered a climate‐sensitive zoonosis because climatic conditions modify pathogen persistence, livestock ecology, and opportunities for human exposure rather than acting as direct causal agents [10, 26]. The interaction of these climatic stressors with traditional livestock systems—such as pastoralism and nomadic herding, which involve close human–animal contact and the consumption of raw animal products—renders HB a climate‐sensitive disease in the region. Drought conditions can concentrate livestock around limited water and forage resources, intensify human–animal interactions, alter herd mobility patterns, and potentially enhance the environmental persistence of Brucella spp., collectively increasing the risk of zoonotic transmission [27].

Despite growing evidence from the Middle East, findings on the association between climatic and environmental factors and HB remain heterogeneous. These inconsistencies likely arise from differences in spatial scale (national vs. subnational analyses), climatic contexts (arid vs. semiarid regions), study designs, and statistical approaches, as well as insufficient adjustment for socioeconomic and husbandry‐related confounders. Moreover, the strong geographic concentration of studies in Iran limits regional comparability and may obscure context‐specific transmission pathways in other Middle Eastern countries. Despite the growing number of primary studies conducted across the Middle East, no previous systematic review has comprehensively synthesized the available evidence on climatic and environmental determinants of HB in this region. Consequently, important knowledge gaps remain regarding the consistency of reported associations, sources of heterogeneity, and the implications of these findings for climate‐informed surveillance and prevention strategies in the Middle East.

In this context, the present systematic review aims to synthesize existing evidence on the effects of climatic and environmental factors on HB in the Middle East. Specifically, we seek to (i) identify and categorize direct and indirect climate‐sensitive determinants of brucellosis, (ii) explain the sources of heterogeneity across studies, and (iii) derive implications for climate‐informed surveillance and prevention strategies in a region highly vulnerable to climate change. Given the complex interactions among climate, livestock, environmental conditions, and human exposure, understanding climate‐sensitive transmission of HB requires a One Health perspective that integrates environmental, animal, and human health dimensions.

## 2. Materials and Methods

### 2.1. Protocol

This systematic review was conducted and reported in accordance with the PRISMA 2020 guidelines and was prospectively registered in the Open Science Framework (OSF) at https://doi.org/10.17605/OSF.IO/75P2T.

### 2.2. Information Sources and Search Strategy

A comprehensive search was performed in Embase, Web of Science, Scopus, and PubMed up to February 9, 2025, with no publication date limits. The search strategy was initially developed for PubMed and subsequently adapted for Embase, Scopus, and Web of Science. The search combined keywords and MeSH terms using Boolean operators (AND, OR) to maximize sensitivity. The search string included the following terms:

(“Middle East” OR “Bahrain” OR “Iran” OR “Iraq” OR “Israel” OR “Jordan” OR “Kuwait” OR “Lebanon” OR “Oman” OR “Qatar” OR “Saudi Arabia” OR “Syria” OR “Turkey” OR “United Arab Emirates” OR “Yemen” OR “Egypt” OR “Palestine”) AND (“Climatic Variables” OR “Climate Change” OR “Environmental Factors” OR “Climate Variability” OR “Climatic Patterns” OR “Seasonal Patterns” OR “Temporal Trends” OR “climate conditions” OR “Climatic Factors” OR temperature OR climate OR precipitation OR humidity OR rainfall OR “Ecological determinants” OR “Climate effect”) AND (“human Brucellosis” OR “Brucella” OR “Brucellosis” OR “undulant fever” OR “Mediterranean fever” OR “Malta fever” OR “Gibraltar fever” OR “thousand face” OR “raging fever” OR “melitococcis”). Additional sources were identified through manual citation tracking (snowballing) and reviewing reference lists of related articles.

### 2.3. Inclusion and Exclusion Criteria

Studies included were original, peer‐reviewed articles published in English that examined the relationship between climatic or environmental variables and HB incidence in Middle Eastern countries. Descriptive, cross‐sectional, analytical, or modeling studies (ARIMA, DLNM, machine learning) were considered. Gray literature such as conference papers, theses, short articles, letters, reviews, or studies without full text or relevant human or environmental data (e.g., temperature, precipitation, humidity, wind speed, evaporation, sunshine hours, frost days, seasons, vegetation cover, altitude, and slope) were excluded. Studies irrelevant to the research objectives or lacking extractable results were also discarded.

### 2.4. Screening Process

Two independent reviewers screened all titles and abstracts for relevance. Full texts of potentially eligible studies were retrieved and assessed, with disagreements resolved through discussion or a third reviewer’s judgment. Titles and abstracts not meeting criteria were excluded, followed by a full‐text review to confirm relevance and adherence to exclusion criteria. Exclusion reasons were documented (Supporting 1), with disagreements resolved via discussion. The study selection process is illustrated in a PRISMA flow diagram.

### 2.5. Quality Assessment and Risk of Bias

The methodological quality and risk of bias of the included studies were assessed using validated, design‐specific appraisal tools to ensure an appropriate and consistent evaluation across heterogeneous study types. Cross‐sectional, time‐series, and quasiexperimental studies were appraised using the Joanna Briggs Institute (JBI) Critical Appraisal Checklists [28], while predictive and forecasting studies—including ARIMA models, ANFIS, and machine learning–based approaches—were evaluated using the TRIPOD checklist, which is specifically designed for prediction model development and validation studies [29]. Case–control and cohort studies were assessed using the Newcastle–Ottawa Scale (NOS) [30]. The use of multiple appraisal tools was necessary to reflect the methodological diversity of the included studies and to ensure that each study design was evaluated using the most appropriate criteria.

All quality assessments were conducted independently by two reviewers, with discrepancies resolved through discussion or consultation with a third reviewer when necessary. For each study, checklist scores were converted into percentage‐based quality ratings and classified as excellent (85%–100%), good (70%–84%), moderate (50%–69%), or poor (< 50%). Overall quality assessments were summarized and reported in the supporting information (Supporting 2 and 3) and were considered in the interpretation of findings, particularly when explaining heterogeneity across studies.

### 2.6. Data Extraction

Data were extracted using a standardized form, including

• Study details (authors, year, and location)
• Study design, sample size, and data collection period
• Climatic and environmental variables
• Geographical region (country)
• Brucellosis outcomes (incidence, prevalence, or model predictions)
• Main climate‐related findings
• Recommended control and prevention strategies

Two reviewers independently performed data extraction. To facilitate the structured narrative synthesis, additional information was extracted on the predominant climatic setting of each study (e.g., arid, semiarid, or humid‐temperate) and methodological approach (observational vs. predictive modeling). These characteristics were subsequently used to organize the synthesis and explore potential sources of heterogeneity across studies.

### 2.7. Data Analysis

Given the substantial methodological and clinical heterogeneity among the included studies—including differences in study design, climatic exposures, outcome definitions, spatial and temporal scales, and statistical approaches—a quantitative meta‐analysis was not considered methodologically appropriate, consistent with recommendations for evidence synthesis in the presence of substantial heterogeneity [31]. Instead, a structured narrative synthesis was undertaken in accordance with established guidance for synthesizing heterogeneous evidence [32].

To improve comparability across studies, the synthesis was organized using multiple complementary frameworks. First, studies were grouped according to the type of climatic or environmental exposure evaluated (e.g., temperature, precipitation, humidity, wind speed, evaporation, seasonal patterns, vegetation cover, elevation, and land slope). Second, studies were categorized according to their predominant climatic setting (arid, semiarid, or humid‐temperate) and methodological approach (observational vs. predictive modeling). These groupings enabled a more systematic exploration of consistency and heterogeneity in reported associations.

For each climatic or environmental variable, the direction of reported findings (positive, negative, or no statistically significant association) was summarized to evaluate the consistency of evidence across studies. For predictive modeling studies, findings were synthesized separately by describing the contribution of climatic variables to model performance rather than interpreting them as epidemiological associations. Where reported, temporal lag structures between climatic exposures and HB incidence were also considered. Finally, findings were interpreted in light of methodological quality, ecological context, and potential sources of heterogeneity rather than relying solely on the statistical significance reported in individual studies.

## 3. Results

### 3.1. Study Identification

**FIGURE 1:**
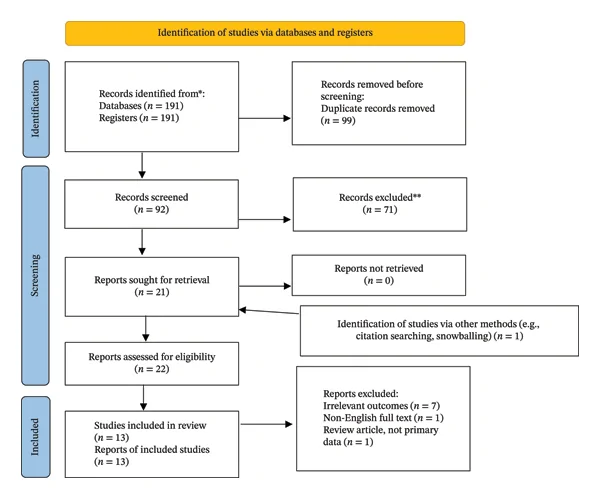
PRISMA flowchart depicting the search, selection, and data extraction process.

**FIGURE 2:**
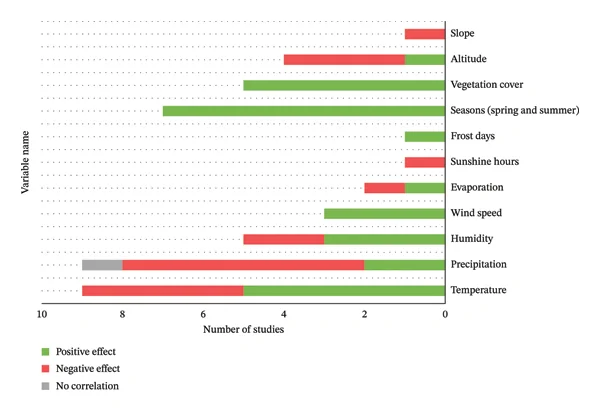
Distribution of studies on climatic and environmental variables affecting human brucellosis incidence in the Middle East by effect type.

The study selection process is illustrated in the PRISMA flow diagram (Figure 1). The frequency with which different climatic and environmental variables were examined across the included studies is presented in Figure 2. A summary of study characteristics and main findings is provided in Table 1, while detailed extracted data are available in Supporting 4. The overall direction and consistency of reported associations between climatic/environmental variables and HB are summarized in Table 2, which served as the basis for the structured narrative synthesis.

**TABLE 1:** Characteristics of included studies and methodological classification used for the structured narrative synthesis.

Study ID	First author (year)	Study design	Study category	Country/region	Climate setting	Climatic and environmental variables	Main climate‐related findings
1	Ahmadkhani et al. (2017)	Cross‐sectional with GIS and statistical modeling	Observational	Iran	Semiarid	Temperature, precipitation, wind speed, NDVI, season	Positive associations with temperature, wind speed, and vegetation; negative association with precipitation; higher incidence during spring and summer.
2	Mustafa et al. (2024)	Retrospective descriptive	Observational	Iraq	Arid to semiarid	NDVI, season	Higher incidence in areas with greater vegetation cover; seasonal peak during spring and summer.
3	Jafarzadeh et al. (2024)	Retrospective cross‐sectional	Observational	Ardabil, Iran	Semiarid	Season	Highest incidence observed between May and August.
4	Rahmanian et al. (2018)	Descriptive‐analytical	Observational	Jahrom, Iran	Arid to semiarid	Temperature, humidity, precipitation	Positive association with temperature; no statistically significant association with precipitation; humidity was reported descriptively without formal association analysis.
5	Rahmanian et al. (2020)	Time‐series (ARIMA)	Predictive modeling	Yazd, Iran	Arid	Temperature, humidity, season	ARIMA model identified clear seasonal variation with higher predicted incidence during March–June; climatic variables were incorporated for forecasting rather than association analysis.
6	Mohammadian‐Khoshnoud et al. (2021)	Time‐series (Markov Switching Model)	Predictive modeling	Qazvin, Iran	Semiarid	Temperature, precipitation, wind speed	Minimum temperature and wind speed improved model prediction, whereas precipitation showed an inverse contribution to predicted incidence.
7	Bagheri et al. (2020)	Machine‐learning time‐series	Predictive modeling	Western Iran	Semiarid to subhumid	Temperature, precipitation, wind speed	Temperature, precipitation, and wind speed were identified as important predictors in forecasting models.
8	Babaie et al. (2021)	GIS‐based ANFIS modeling	Predictive modeling	Mazandaran, Iran	Humid–temperate	Temperature, precipitation, humidity, evaporation, NDVI	Prediction model suggested negative effects of temperature and precipitation, positive effects of humidity and vegetation, and a weak positive effect of evaporation on predicted disease occurrence.
9	Kanannejad et al. (2016)	Cross‐sectional	Observational	Kohgiluyeh and Boyer‐Ahmad, Iran	Semiarid	Temperature, rainfall, elevation, slope	Lower rainfall, lower elevation, and lower land slope were associated with higher disease occurrence.
10	Faramarzi et al. (2019)	Cross‐sectional	Observational	Fars, Iran	Semiarid	Temperature, rainfall, humidity, evaporation, sunshine hours, frost days	Negative association with temperature and sunshine duration; positive associations with rainfall, humidity, and frost days.
11	Shirzadi et al. (2018)	National ecological study	Observational	Iran	Multiple climatic zones	Temperature, rainfall, vegetation, elevation	Negative associations with temperature and elevation; positive associations with rainfall and vegetation.
12	Al‐Awaidy & Al‐Hashami (2017)	Descriptive‐analytical	Observational	Oman	Arid	Season, precipitation, vegetation, elevation	Higher incidence associated with precipitation, vegetation, and elevation; seasonal peak during spring and summer.
13	Al‐Shehhi et al. (2016)	Surveillance data analysis	Observational	Abu Dhabi, UAE	Arid	Season	Higher incidence reported during warmer months.

*Note:* Climate setting was assigned according to the predominant climatic characteristics of each study area to facilitate structured narrative synthesis and comparison across studies. Predictive modeling studies evaluated the contribution of climatic variables to forecasting performance and should not be interpreted as demonstrating epidemiological causality or independent associations.

Abbreviations: ANFIS, adaptive neurofuzzy inference system; ARIMA, autoregressive integrated moving average; GIS, geographic information system; NDVI, Normalized Difference Vegetation Index.

**TABLE 2:** Direction and consistency of reported associations between climatic and environmental variables and human brucellosis in the Middle East.

Variable	No. of studies	Positive	Negative	No significant association	Overall consistency
Temperature	9	5	4	0	Moderate
Precipitation/rainfall	9	2	6	1	Moderate
Relative humidity	5	3	2	0	Low
Wind speed	3	3	0	0	High
Evaporation	2	1	1	0	Inconsistent
Sunshine hours	1	0	1	0	Limited evidence
Frost days	1	1	0	0	Limited evidence
Seasonal pattern	7	7	0	0	High
Vegetation (NDVI)	5	5	0	0	High
Elevation	4	1	3	0	Moderate
Land slope	1	0	1	0	Limited evidence

*Note:* Overall consistency was determined based on the proportion of studies reporting associations in the same direction and should not be interpreted as the certainty or strength of evidence.

### 3.2. Methodological Quality

**FIGURE 3:**
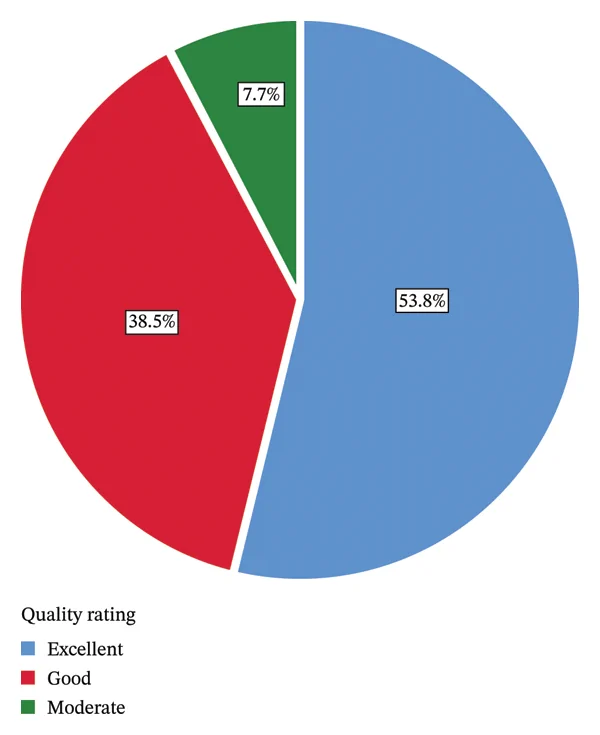
Pie chart showing the distribution of studies by methodological quality.

Among the 13 included studies, seven (53.8%) were rated as “excellent,” reflecting strong methodological rigor, transparent reporting, and appropriate analytical approaches. Five studies (38.5%) were rated as “good,” commonly due to limited adjustment for potential confounders or incomplete reporting of missing data. One study (7.7%) was classified as “moderate” (Figure 3).

**FIGURE 4:**
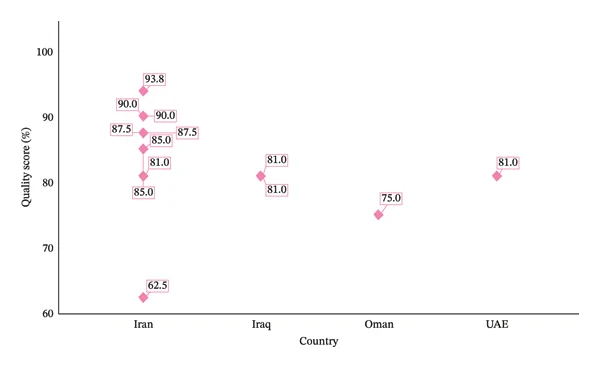
Stacked bar chart displaying study distribution by region and methodological quality.

Most studies were conducted in Iran, while fewer investigations were performed in other Middle Eastern countries such as Saudi Arabia, Jordan, Syria, Oman, and the United Arab Emirates (Figure 4). High‐quality studies more consistently identified statistically significant associations between climatic variables—particularly temperature, precipitation, humidity, and seasonal patterns—and HB incidence. In contrast, studies with lower methodological quality or limited adjustment for potential confounding variables tended to report more heterogeneous findings, highlighting the importance of methodological rigor when interpreting climate–disease relationships. The predominance of observational studies conducted in arid and semiarid regions, together with the inclusion of several predictive modeling studies, provided the basis for the structured narrative synthesis presented in the following sections (Table 1).

Overall, the included studies demonstrated considerable heterogeneity in the reported associations between climatic factors and HB. This variability reflected differences in climatic settings, geographical scale, methodological approaches, and statistical adjustment strategies. Nevertheless, several consistent patterns emerged across studies. Temperature, precipitation, and humidity were the most frequently investigated variables, while seasonal patterns and vegetation cover showed the highest consistency in their associations with disease incidence. Predictive modeling studies generally demonstrated higher consistency in identifying climate‐sensitive predictors than observational studies, although epidemiological evidence remained more heterogeneous.

### 3.3. Overview of the Evidence Synthesis

Overall, the included studies demonstrated considerable methodological and contextual heterogeneity. Variations were observed in climatic settings, geographical scales, study designs, statistical approaches, and the climatic and environmental variables investigated. Most studies were conducted in arid or semiarid settings, reflecting the predominant ecological conditions of the Middle East, whereas only one study was undertaken in a humid–temperate region (Mazandaran Province, northern Iran). To facilitate a structured narrative synthesis, studies were categorized according to climatic setting, methodological approach (observational vs. predictive modeling), and the climatic and environmental variables examined. The methodological characteristics of the included studies are summarized in Table 1, whereas the overall direction and consistency of reported climate–brucellosis relationships are presented in Table 2.

### 3.4. Climatic Variables

Temperature was the most frequently investigated climatic variable, examined in nine of the 13 included studies. Five studies reported a positive association between increasing temperature and HB incidence [33–37], whereas four identified an inverse relationship [12, 38–40]. One study further demonstrated a nonlinear association, with incidence increasing at moderate temperatures but declining at temperature extremes [35]. Overall, these findings suggest moderate consistency regarding the influence of temperature; however, the direction of the association varied across climatic settings and analytical approaches, indicating that temperature may influence transmission through context‐dependent ecological mechanisms rather than exerting a uniform effect.

Precipitation was assessed in nine studies. Two studies reported a positive association with HB incidence [40, 41], six studies reported a negative association [12, 33–35, 38, 39], and one study found no statistically significant relationship [37]. The predominance of inverse associations suggests that reduced precipitation may increase opportunities for transmission in arid environments, although contradictory findings indicate that local ecological and livestock management conditions likely modify this relationship.

Humidity was examined in five studies. Three studies reported a positive association with disease incidence [12, 38, 40], while two studies reported a negative association [36, 37]. Overall, evidence regarding humidity was inconsistent, suggesting that its effects may depend on interactions with temperature, rainfall, and local environmental conditions rather than acting as an independent climatic driver.

Wind speed was evaluated in three studies and consistently showed a positive indirect association with HB incidence [33–35]. Although evidence was limited, the consistent findings across studies suggest that wind speed may indirectly contribute to disease transmission through environmental and ecological pathways.

Evaporation was examined in two studies, with one reporting a positive association [38] and the other reporting a negative association [12]. Sunshine hours and frost days were each examined in one study, revealing a negative association with sunshine duration and a positive association with frost days and HB incidence [12].

### 3.5. Environmental Variables

Seasonal patterns were examined in seven studies, with consistently higher HB incidence reported during spring and summer and lower incidence during autumn and winter [33, 36–38, 42–44]. Seasonal variation represented the most consistent environmental determinant across the included studies, with all seven investigations reporting peak incidence during spring and summer.

Vegetation cover was analyzed in five studies, all of which reported a positive indirect association with HB incidence [33, 38, 40, 41, 44].

Elevation was examined in four studies. Three studies reported a negative association between higher altitude and disease incidence [38–40], while one study reported a positive association [41].

Land slope was assessed in one study and demonstrated a negative indirect association with HB incidence [39].

## 4. Discussion

This systematic review demonstrates that climatic and environmental factors play a substantial role in shaping the incidence and spatial–temporal distribution of HB in the Middle East. However, the direction and magnitude of these effects vary considerably across settings, reflecting regional climatic heterogeneity, differences in livestock management systems, human behavioral patterns, and the methodological diversity of the included studies. These findings highlight the multifactorial and context‐dependent nature of climate–brucellosis interactions and underscore the need for integrated interpretations rather than single‐variable explanations.

### 4.1. Causality Considerations

Although the included studies consistently identified associations between climatic and environmental factors and HB (HB), these findings should not be interpreted as evidence of direct causality. Most of the included studies employed ecological, cross‐sectional, retrospective, or time‐series designs, which are appropriate for identifying population‐level associations and temporal patterns but are inherently limited in their ability to establish causal relationships. Rather than acting as independent causal determinants, climatic variables are more likely to influence HB indirectly by modifying ecological and behavioral pathways, including livestock reproductive cycles, grazing patterns, environmental persistence of Brucella spp., and the frequency of human–animal interactions [45, 46].

Interpretation of these findings should also consider the potential for ecological fallacy, whereby associations observed using aggregated climatic and surveillance data may not accurately reflect individual‐level risks or causal mechanisms. Because most studies analyzed data at provincial, regional, or national levels, caution is warranted when extrapolating population‐level associations to individual exposures [47].

In addition, several important factors that may modify or confound the relationship between climate and HB—including livestock vaccination coverage, animal movement, herd management practices, consumption of unpasteurized dairy products, livestock density, veterinary control programs, animal vaccination programs, socioeconomic conditions, and the completeness of disease surveillance—were inconsistently measured or adjusted for across the included studies. These unmeasured or residual confounders may partly explain the heterogeneity observed between studies and highlight the need for future research integrating climatic, epidemiological, veterinary, and socioeconomic data within a One Health framework [45, 46].

### 4.2. Climatic Variables

Temperature was the most frequently investigated climatic factor and showed heterogeneous effects on HB incidence. Five studies reported a positive association between increasing temperature and HB incidence [33–37], suggesting that moderate‐to‐high temperatures may enhance Brucella survival in the environment and increase human exposure through intensified livestock activity and consumption of unpasteurized animal products. Experimental evidence supports this hypothesis, indicating that Brucella species can remain viable under warm conditions, particularly in organic substrates such as soil, manure, and dairy products [48].

In contrast, four studies reported an inverse relationship between temperature and HB incidence [12, 38–40]. These discrepancies may reflect regional climatic thresholds beyond which extreme heat reduces environmental humidity, limits bacterial persistence, or alters human and livestock behaviors, such as reduced outdoor activity or improved food hygiene practices [11, 18, 49, 50]. One study further reported a nonlinear relationship, with increasing incidence at moderate temperatures and declining incidence at extreme values [35], highlighting the importance of threshold effects and interactions with other climatic variables.

Precipitation also showed complex and context‐dependent associations with HB. While two studies reported a positive relationship [40, 41], six studies found a negative association [12, 33–35, 38, 39], and one study reported no significant effect [37]. Moderate rainfall may facilitate Brucella persistence by maintaining soil moisture and vegetation, indirectly increasing livestock–human contact [18]. Conversely, heavy rainfall or flooding may reduce transmission by washing away contaminated materials and limiting direct exposure [22, 51, 52]. In arid or semiarid regions, drought conditions can intensify transmission by forcing livestock and humans to congregate around limited water sources, as documented in pastoral settings in East Africa [53, 54].

Humidity exhibited a similarly dual role. Three studies reported a positive association with HB incidence [12, 38, 40], likely due to enhanced bacterial survival in humid environments, where Brucella can persist for extended periods in water, manure, and organic matter [54–56]. In contrast, two studies reported a negative association [36, 37], particularly in dry regions where low humidity may increase livestock aggregation and human contact around scarce resources [57, 58]. These findings suggest a nonlinear relationship modulated by livestock density, farming practices, and local climate.

Wind speed consistently showed an indirect but significant role in HB transmission [33–35], likely through the dispersion of contaminated dust and aerosols in rural and pastoral areas. Evidence from northwest China supports this mechanism, demonstrating lagged associations between wind speed and HB incidence [22]. Predictive models, including SARIMAX and machine‐learning approaches, further highlight wind speed as a key contributor to outbreak forecasting [18, 59].

Evaporation showed contradictory effects. A positive association was reported in one study [38], suggesting that higher evaporation may promote airborne transmission via dust dispersion, whereas another study found a negative effect, possibly due to extreme dryness limiting bacterial survival [12]. Globally, evaporation may influence HB transmission through multiple pathways, including altered surface humidity, livestock movement, and environmental disruption under severe drought [14, 60].

Sunshine duration and frost days were less frequently studied but appear to act as climatic modifiers. Reduced HB incidence with longer sunshine hours may reflect ultraviolet radiation reducing Brucella viability [12, 22], although contrasting findings suggest that increased grazing activity during sunny periods may elevate risk in some regions [13, 14]. Frost days were positively associated with HB in one study [12], potentially due to prolonged bacterial survival in cold environments, although other studies suggest reduced transmission during cold periods because of decreased human–livestock interaction [13, 61, 62].

Seasonal analyses consistently identified spring and summer as peak transmission periods [33, 36–38, 42–44], coinciding with livestock birthing seasons, increased grazing, and higher human–animal contact. These patterns align with global observations from Asia and Africa [13, 14, 22, 42, 63] and may be altered under future climate change scenarios.

Overall, the findings of this review are broadly consistent with evidence from other endemic regions, including China, Central Asia, the Mediterranean Basin, and East Africa, where temperature, precipitation, seasonal variation, and vegetation have likewise been identified as important determinants of HB transmission [13, 14, 18, 22, 42, 53, 54]. However, important regional differences were also evident. In the Middle East, climate appears to influence transmission primarily through its interaction with traditional livestock husbandry, pastoral and nomadic production systems, and prolonged human–animal contact. In contrast, studies from China and Central Asia have more frequently emphasized the contribution of large‐scale livestock density, grazing patterns, and environmental suitability, whereas research from East Africa has highlighted the role of drought‐driven livestock aggregation and water scarcity. These observations indicate that climatic variables operate through context‐specific ecological and socioeconomic pathways rather than exerting uniform effects across endemic regions.

### 4.3. Environmental Variables

Vegetation cover consistently showed a positive indirect association with HB incidence [33, 38, 40, 41, 44], likely by shaping livestock habitats, grazing intensity, and pathogen persistence. Similar associations have been reported in China, where higher NDVI values were linked to increased HB risk through enhanced livestock concentration and environmental suitability [18, 22, 48, 64, 65].

Altitude showed mixed effects. While three studies reported a negative association with HB incidence [38–40], one study from Oman reported a positive relationship [41]. These discrepancies likely reflect regional climatic gradients and livestock systems, as high‐altitude areas may offer favorable ecological niches for Brucella in some contexts while limiting transmission in others [66–69].

Land slope was examined in only one study, which reported a negative association with HB incidence [39]. Steeper slopes may reduce livestock density and pathogen persistence due to improved drainage and less favorable grazing conditions [70]. However, evidence from other regions suggests that slope interacts with humidity, soil type, and vegetation, potentially creating microenvironments conducive to bacterial survival [18, 71, 72].

### 4.4. Predictive Versus Mechanistic Evidence

An important distinction emerging from this review is the difference between predictive modeling studies and epidemiological investigations. Predictive modeling studies included in this review—including ARIMA, ANFIS, and machine‐learning approaches—demonstrated that climatic variables can improve forecasting performance and may provide useful tools for climate‐informed early warning systems [34, 36, 38]. However, predictive importance should not be interpreted as evidence of biological or epidemiological causation [73, 74]. In contrast, observational studies provide evidence regarding associations between climatic factors and disease occurrence but remain susceptible to residual confounding and ecological bias [47]. Therefore, predictive and observational findings should be regarded as complementary rather than interchangeable sources of evidence for understanding climate‐sensitive transmission of HB [74].

Overall, these findings gain additional importance in the context of ongoing climate change, which is expected to alter temperature regimes, precipitation patterns, and ecological conditions across the Middle East. As climate‐sensitive transmission pathways become increasingly dynamic, surveillance systems should integrate climatic information with veterinary and public health data to support early warning, risk mapping, and targeted prevention strategies. Such integrated approaches are consistent with the One Health framework and may improve preparedness for future climate‐related shifts in HB transmission.

## 5. Conclusion

This systematic review demonstrates that HB in the Middle East is strongly influenced by climatic and environmental determinants operating through interconnected human–animal–environment pathways. Direct climatic drivers, particularly temperature and precipitation, appear to influence disease transmission by modifying the environmental persistence of Brucella spp., livestock behavior, and patterns of human exposure, while environmental factors such as vegetation cover, altitude, and land slope act indirectly by shaping ecological niches, grazing systems, and human–livestock interactions. The heterogeneity observed across the included studies indicates that climate–brucellosis relationships are highly context‐dependent and mediated by regional ecological conditions, livestock management practices, and public health capacity. Taken together, these findings support the characterization of HB as a climate‐sensitive zoonotic disease in the Middle East while emphasizing that current evidence is derived predominantly from observational studies and should therefore be interpreted with appropriate caution. Integrating climatic, environmental, veterinary, and public health information within a One Health framework may strengthen climate‐informed surveillance, early warning systems, and evidence‐based prevention strategies in this highly vulnerable region.

## 6. Limitations and Future Directions

The interpretation of these findings should consider several important limitations. First, the available evidence was heavily concentrated in Iran, limiting the geographical representativeness of the review and restricting the generalizability of findings to the wider Middle East. Second, substantial methodological heterogeneity existed across studies with respect to study design, climatic variables, spatial and temporal scales, statistical approaches, and outcome definitions, making direct comparisons challenging.

Furthermore, because most included studies employed ecological, cross‐sectional, or retrospective designs, population‐level associations should not be interpreted as evidence of individual‐level causal relationships. Several important factors—including socioeconomic conditions, livestock vaccination coverage, animal movement, consumption of unpasteurized dairy products, veterinary control programs, livestock density, and surveillance quality—were also inconsistently measured or adjusted for, potentially contributing to residual confounding and between‐study heterogeneity. In addition, the inclusion of both observational and predictive modeling studies strengthened the overall evidence base but also introduced methodological diversity, as predictive performance should not be interpreted as equivalent to epidemiological or biological causation.

Future research should prioritize underrepresented countries across the Middle East, adopt standardized epidemiological protocols together with transparent reporting of predictive modeling studies, and integrate long‐term climatic, environmental, veterinary, and epidemiological datasets. Greater attention should also be given to indirect climate‐sensitive mechanisms—including changes in vegetation, water availability, livestock mobility, land use, and human behavior—that may mediate transmission pathways. Strengthening regional collaboration, harmonizing surveillance systems, and promoting data sharing within a One Health framework will be essential for generating more robust evidence and supporting climate‐resilient surveillance, early warning systems, and coordinated brucellosis prevention and control strategies throughout the region.

## Author Contributions

Aboozar Soltani and Marziyeh Hamyali‐Ainvand designed and conceptualized the study. Kourosh Azizi, Mohammad Ebrahimi, and Marziyeh Hamyali‐Ainvand gathered the data, and Marziyeh Hamyali‐Ainvand and Aboozar Soltani analyzed them. Marziyeh Hamyali‐Ainvand, Kourosh Azizi, and Aboozar Soltani drafted the manuscript. All the authors participated in writing the manuscript.

## Funding

This research received no specific grant from any funding agency in the public, commercial, or not‐for‐profit sectors.

## Disclosure

All authors read and approved the final manuscript.

## Ethics Statement

This study is a systematic review and did not involve human participants or personal data, so ethical approval was not required.

## Conflicts of Interest

The authors declare no conflicts of interest.

## Supporting Information

Additional supporting information can be found online in the Supporting Information section.

## Supporting information


**Supporting Information** Supporting 1 details the reasons for exclusion at the full‐text screening stage. Supporting 2 and 3 present the complete quality assessment results of included studies. Supporting 4 provides the detailed extracted data for all eligible studies, complementing the summary of study characteristics and main findings reported in Table 1.

## Data Availability

All data generated or analyzed during this study are included in this published article and its supporting information files.
