# Supplementary material for: Climate‐Sensitive Transmission of Human Brucellosis: A Systematic Review of Climatic and Environmental Determinants in the Middle East
Source: J Trop Med. 2026 Aug 30;2026:8884033. doi: 10.1155/jotm/8884033 (PMC13526250; doi:10.1155/jotm/8884033)
Supplement: Supplementary file 1 [file JOTM-2026-8884033-s001.zip › Supplementary 1.pdf]

Supplementary File 1. Search returns that were excluded after full-text screening and the reason for exclusion

Authors	Title of search return	Reason for exclusion
Todd EC, 2017	Foodborne disease and food control in the Gulf States	Irrelevant outcomes
Aljanazreh B et al., 2022	Brucellosis re-emergence after a decade of quiescence in Palestine, 2015–2017: A seroprevalence and molecular characterization study	Irrelevant outcomes
Bora G et al., 2016	Investigation of Brucella seroprevalence in human and livestocks in Igdır, Turkey	Irrelevant outcomes
Hedayati MA, Bahmani N, 2023	Most important bacterial and parasitic zoonotic diseases in Iran	Review article, not primary data
Herzallah HK et al , 2019	Temporal trends in the incidence and demographics of cancers, communicable diseases, and non-communicable diseases in Saudi Arabia over the last decade	Irrelevant outcomes
Pordanjani SR et al., 2024	Spatial Epidemiology and Temporal Trend of Brucellosis in Iran Using Geographic Information System (GIS) and Join Point Regression Analysis: An Ecological 10-Year Study	Irrelevant outcomes
Jafari A et al., 2024	Spatial prediction of human brucellosis susceptibility using an explainable optimized adaptive neuro fuzzy inference system	Irrelevant outcomes
Tapak L et al., 2018	Predicting the Frequency of Human Brucellosis using Climatic Indices by Three Data Mining Techniques of Radial Basis Function, Multilayer Perceptron and Nearest Neighbor: A Comparative Study	Non-English full text
Sulaiman SO et al., 2018	Precipitation pattern modeling using cross-station perception: regional investigation	Irrelevant outcomes
