# Supplementary material for: Climate‐Sensitive Transmission of Human Brucellosis: A Systematic Review of Climatic and Environmental Determinants in the Middle East
Source: J Trop Med. 2026 Aug 30;2026:8884033. doi: 10.1155/jotm/8884033 (PMC13526250; doi:10.1155/jotm/8884033)
Supplement: Supplementary file 1 [file JOTM-2026-8884033-s001.zip › Supplementary 2.docx.pdf]

Supplementary File 2: Quality Assessment of Studies on Human Brucellosis in the Middle East: A Summary of Key Findings and Limitations

No.	Study Title	Study Type	Quality Assessment Checklist	Final Score (%)	Quality Rank	Key Points/Limitations
**1**	Space-time analysis of human brucellosis considering environmental factors in Iran	Cross-sectional	JBI	81%	Good	Lack of control over socio-economic variables
**2**	Epidemiological Characteristics and Temporal Trend of Human and Bovine Brucellosis Cases in Southern Iran	Descriptive-Analytical	JBI	62.5%	Moderate	Inadequate sample selection specification
**3**	Time-Series Analysis and Forecasting of Human Brucellosis in Yazd Province Using ARIMA Model	Time-Series	JBI	87.5%	Excellent	Lack of control over confounding variables
**4**	Predicting the incidence of brucellosis in Western Iran using Markov switching model	Time-Series	JBI	93.75%	Excellent	Inadequate control over confounding variables
**5**	Forecasting the monthly incidence rate of brucellosis in west of Iran using time series and data mining	Time-Series	TRIPOD	90%	Excellent	Unspecified management of missing data
**6**	Spatial prediction of human brucellosis using GIS-based ANFIS	Model-Based	TRIPOD	90%	Excellent	No explanation on missing data
**7**	Effect of human, livestock population, climatic and environmental factors on brucellosis in Southwest Iran	Cross-sectional	JBI	87.5%	Excellent	Lack of control over socio-economic variables

**8**	Potential Effects of Climatic Parameters on Human Brucellosis in Fars Province	Model-Based	TRIPOD	85%	Excellent	Unspecified ratio of training/testing data
**9**	The Incidence and Geographical Distribution of Brucellosis in Iran Using GIS	Cross-sectional	JBI	75%	Good	Inadequate control over confounding variables
**10**	Spatial Distribution and Epidemiological Factors of Brucellosis in Ardabil Province	Cross-sectional	JBI	81%	Good	Failure to investigate socio-economic factors
**11**	Zoonotic Diseases in Oman: Successes and Challenges	Descriptive-Analytical	JBI	75%	Good	Lack of control over socio-economic variables
**12**	Human Brucellosis in Iraq: Spatiotemporal Data Analysis	Cross-sectional	JBI	81%	Good	Lack of control over confounding variables
**13**	Human brucellosis in Abu Dhabi, UAE	Cross-sectional	JBI	81%	Good	Absence of social data and limited laboratory tests
