# Supplementary material for: Climate‐Sensitive Transmission of Human Brucellosis: A Systematic Review of Climatic and Environmental Determinants in the Middle East
Source: J Trop Med. 2026 Aug 30;2026:8884033. doi: 10.1155/jotm/8884033 (PMC13526250; doi:10.1155/jotm/8884033)
Supplement: Supplementary file 1 [file JOTM-2026-8884033-s001.zip › Supplementary 3.docx.pdf]

Supplementary File 3. A summary of the quality appraisal results by JBI, TRIPOD

1.JBI

Study	Were inclusion and exclusion criteria clearly defined?	Was the study population clearly described?	Were confounding factors identified and controlled for?	Were the measurement methods valid and reliable?	Was appropriate statistical analysis used?	Were the results clearly reported?	Were causal relationships appropriately interpreted?	Were study limitations reported?
(Shirzadi et al. 2021)	Partially	Yes	No	Yes	Yes	Yes	Yes	Yes
(Rahmanian et al. 2018)	Yes	Yes	No	Yes	Partially	Partially	Partially	Yes
(Mustafa et al. 2024)	Yes	Yes	No	Yes	Yes	Yes	Partially	Yes
(Al Shehhi et al. 2016)	Yes	Yes	No	Yes	Yes	Yes	Partially	Yes
(Jafarzadeh et al. 2024)	Yes	Yes	No	Yes	Yes	Yes	Partially	Yes
(Ahmadkhani et al. 2017)	Yes	Yes	No	Yes	Yes	Yes	Partially	Yes
(Awaidy et al. 2017)	Yes	Yes	No	Yes	Yes	Yes	Partially	Yes
(Rahmanian et al. 2020)	Yes	Yes	No	Yes	Yes	Yes	Yes	Yes
(Mohammadian Khoshnoud et al. 2021)	Yes	Yes	Partially	Yes	Yes	Yes	Yes	Yes

(KananNejad et al. 2016)	Yes	Yes	Partially	Yes	Yes	Yes	Partially	Yes

2. TRIPOD

Study	Is the objective and intended use of the predictive model clearly stated?	Is the study population and the data used for model development clearly defined?	Are the predictor variables explicitly described and clearly introduced?	Is the sampling method and the process of data splitting into training and testing sets adequately explained?	Is the handling of missing data appropriately described?	Are the statistical modeling methods clearly reported and justified?	Has internal validation of the model (e.g., cross-validation) been conducted?	Are the model results reported with appropriate measures of goodness of fit?	Are the limitations of the model adequately discussed??	Are the study conclusions consistent with the model findings?
(Bagheri et al. 2020)	Yes	Yes	Yes	Yes	No	Yes	Yes	Yes	Yes	Yes
(Babaie et al. 2021)	Yes	Yes	Yes	Yes	No	Yes	Yes	Yes	Yes	Yes
(Faramarzi et al. 2019)	Yes	Yes	Yes	Partially	No	Yes	Yes	Yes	Yes	Yes
